# A Prototype Tool to Enable Farmers to Measure and Improve the Welfare Performance of the Farm Animal Enterprise: The Unified Field Index

**DOI:** 10.3390/ani4030446

**Published:** 2014-07-15

**Authors:** Ian G. Colditz, Drewe M. Ferguson, Teresa Collins, Lindsay Matthews, Paul H. Hemsworth

**Affiliations:** 1 CSIRO Animal, Food and Health Sciences, FD McMaster Laboratory, Armidale, NSW 2350, Australia; E-Mail: Drewe.ferguson@csiro.au; 2 College of Veterinary Medicine, Murdoch University, Murdoch, WA 6150, Australia; E-Mail: T.Collins@murdoch.edu.au; 3 Lindsay Matthews & Associates Research International, Scerne Di Pineto, Teramo 64025, Italy; E-Mail: lindsay.matthews1@gmail.com; 4 Psychology Department, University of Auckland, Auckland 1020, New Zealand; 5 Animal Welfare Science Centre, The University of Melbourne, Parkville, VIC 3010, Australia; E-Mail: phh@unimelb.edu.au

**Keywords:** animal welfare, assessment, unified field index, benchmarking, welfare performance

## Abstract

**Simple Summary:**

Benchmarking is a tool widely used in agricultural industries that harnesses the experience of farmers to generate knowledge of practices that lead to better on-farm productivity and performance. We propose, by analogy with production performance, a method for measuring the animal welfare performance of an enterprise and describe a tool for farmers to monitor and improve the animal welfare performance of their business. A general framework is outlined for assessing and monitoring risks to animal welfare based on measures of animals, the environment they are kept in and how they are managed. The tool would enable farmers to continually improve animal welfare.

**Abstract:**

Schemes for the assessment of farm animal welfare and assurance of welfare standards have proliferated in recent years. An acknowledged short-coming has been the lack of impact of these schemes on the welfare standards achieved on farm due in part to sociological factors concerning their implementation. Here we propose the concept of welfare performance based on a broad set of performance attributes of an enterprise and describe a tool based on risk assessment and benchmarking methods for measuring and managing welfare performance. The tool termed the Unified Field Index is presented in a general form comprising three modules addressing animal, resource, and management factors. Domains within these modules accommodate the principle conceptual perspectives for welfare assessment: biological functioning; emotional states; and naturalness. Pan-enterprise analysis in any livestock sector could be used to benchmark welfare performance of individual enterprises and also provide statistics of welfare performance for the livestock sector. An advantage of this concept of welfare performance is its use of continuous scales of measurement rather than traditional pass/fail measures. Through the feedback provided via benchmarking, the tool should help farmers better engage in on-going improvement of farm practices that affect animal welfare.

## 1. Introduction

Recent decades have seen an increase in societal interest in the management and care of animals used to produce food and fiber. Concern about the quality of life of farm animals has led to an increasing onus on farmers and on food and fiber supply chains to demonstrate that the physical and mental needs of animals are satisfied. In response, a large body of science has addressed criteria for assessing the welfare of animals. Underpinning these issues of quality of life and welfare assessment are philosophical and ethical questions about the relationships between humans and animals. These underpinning issues influence what we understand animal welfare to be and what needs to be measured or assessed in order to draw inferences about the welfare status of farm animals [1].

Three common perspectives can be recognized amongst the many approaches that have been developed to describe and measure animal welfare [2,3,4]. These perspectives can be summarized as understanding and interpreting welfare in terms of:

- Biological functioning—as evidenced through measures of normality of behavioral, physiological, health, and production variables;
- Emotional states—as evidenced through measures of abnormal behaviors, affective states (positive and negative feelings), cognitive function, and suffering.
- Naturalness—as evidenced by attributes of the animal, in particular normal behavioral repertoires, and by attributes of its environment, in particular congruence between the production environment and a notional ideal environment for the species or class of animal.

An important feature of these perspectives for understanding animal welfare is that they provide multiple dimensions for describing welfare in terms of the animal’s status and the environment it experiences. A comprehensive welfare assessment system is therefore likely to need measures that inform each of these perspectives. Definitions of animal welfare developed in recent years, such as the one recently endorsed by the 172 member countries of the office international des epizooties (OIE) [5], encompass aspects of these three perspectives: “Animal welfare means how an animal is coping with the conditions in which it lives. An animal is in a good state of welfare if (as indicated by scientific evidence) it is healthy, comfortable, well nourished, safe, able to express innate behaviour, and if it is not suffering from unpleasant states such as pain, fear, and distress. Good animal welfare requires disease prevention and veterinary treatment, appropriate shelter, management, nutrition, humane handling and humane slaughter/killing. Animal welfare refers to the state of the animal; the treatment that an animal receives is covered by other terms such as animal care, animal husbandry, and humane treatment”.

There are generic challenges to choosing the measures to include in assessment systems that are not unique to animal welfare. For instance, in the design of methods for assessing agricultural sustainability, Binder *et al.* [6] have noted that assessment models should aim for parsimony and sufficiency, and account for interactions between indicators used in the model. Thus, simplicity in the type and number of measures included in an index (parsimony) needs to be balanced against adequacy of the chosen measures to capture critical information needed to make a valid assessment (sufficiency). These design requirements for an effective sustainability assessment tool apply equally to assessment of animal welfare. Thus parsimony and sufficiency can be considered important attributes of an efficient animal welfare assessment system.

The key elements of a welfare assessment system that emerge from the above considerations include:

- The conceptual and ethical perspectives for understanding and describing welfare
- The purpose for undertaking the welfare assessment
- The process for implementing and evaluating the assessment procedure.

In this paper, we describe an assessment tool for use by farmers to manage and improve the welfare performance of their farm animal enterprise. The tool is designed to be refined and managed by farm animal industries. It uses data from farm records to generate industry wide statistics on variables relating to animal welfare. It encourages farmers to consider many of these variables to be performance criteria that, analogous to production criteria like milk yield or growth rate, can be measured on a continuous scale and improved upon from year to year. It harnesses the experience of high performing farmers to identify strategies for ongoing improvement in these variables of welfare performance. It places ownership of welfare performance in the hands of farmers. It differentiates welfare performance measured via the tool from welfare standards implemented through welfare assessment schemes to ensure compliance with regulatory standards. Before describing the tool in more detail and addressing the specific purposes of the tool, we need to consider more generally the range of purposes for which welfare assessments are undertaken.

## 2. Purposes of Welfare Assessment

The need to apply objective methods to assess the welfare status of farm animals usually arises in one of three settings, which align to three specific purposes:

- *Compliance*: To determine compliance with policy, law, and regulatory standards;
- *Market assurance*: To assure both consumers and non-consumers that certain aspects of the welfare of animals, especially those not articulated within 1, are being met, for instance freedom to roam in an outdoor environment or absence of suffering;
- *Welfare management*: To assist owners and managers to monitor and improve the welfare of their livestock

These purposes can be summarized as compliance, market assurance, and welfare management. In fulfilling these purposes the assessment procedures require validity of the measures as indicators of welfare, congruence between the measures and the aspects of welfare under appraisal, and integrity of the assessment procedure. Schemes designed to address compliance and market assurance have a very high dependence on measures that have been validated through research or through processes that generate a consensus of expert opinion. The external focus of compliance and market assurance schemes towards the needs of regulators and consumers means the outcome of assessment is usually graded into various categories such as acceptable or unacceptable. A risk of presenting the outcome of assessment in this way is the failure to provide appropriate feedback, direction or incentive for farmers to continually improve the welfare status of animals on their farms (see [7] for a discussion of this dilemma). Thus, compliance and market assurance schemes might measure welfare status but not provide clear directions on how to improve animal welfare.

These limitations have been revealed in a number of studies. The impact of the Royal Society for the Prevention of Cruelty to Animals (RSPCA) Freedom Food scheme on the welfare of dairy cattle was studied and outcomes of measures compared between farms belonging to the Freedom Food scheme or other schemes [8]. The Freedom Food farms performed worse on some measures including hock injuries, lameness, and restrictions in rising behavior, but better on measures of mastitis, cleanliness, and body condition. Importantly, regardless of the scheme, welfare problems remained prevalent indicating that measuring compliance alone is insufficient to ensure good welfare [9]. The Scottish Agricultural College [10] compared the welfare of dairy cows in organic and conventional milk production systems and showed that levels of lameness and hock damage were lower on organic farms as a result of shorter winter housing periods and a higher age of first calving heifers which are both elements of the less intensive housing approach described in organic standards. Thus, while compliance with specific standards in farm assurance schemes may lead to some improvement in animal welfare [9] there is little compelling evidence that such schemes produce comprehensive or continuous advances in welfare.

### 2.1. The Need for Tools to Assist Farmers Manage and Improve Animal Welfare

The inadequacy of welfare assessment procedures that address compliance and market assurance purposes to lead to welfare improvements points to the need for a separate set of assessment tools to assist farmers in this task. Recognition of the need for such tools is not new and a number of well-developed aids are available to help farmers manage specific welfare and health risks. An example of the detail provided by such aids can be found in the cattle heat load toolbox [11] which enables managers of commercial beef cattle feedlots in Australia to use a combination of input and output measures to predict and manage heat stress events. By using a combination of observed local climatic conditions and animal responses to heat such as panting scores, feedlot managers can manage risks and implement strategies to reduce the impact of severe hot weather [12]. Additional indices, namely the heat load index (HLI) and the accumulated heat load (AHL) determine the animals’ heat load balance taking into account the duration of daily heat exposure and the availability of natural cooling at night [12]. Using a web-based model that incorporates the HLI and AHL indices, feedlot managers can determine specific heat risk assessments for different cattle genotypes on a daily and pen-by-pen basis if required. This tool combines the well quantified physiological impact of heat stress on beef cattle with environmental data to establish criteria for intervention; however, as the impact of heat stress on the animal’s experience and its affective state remain unknown [13], the extent to which the tool addresses the impact of heat load on the emotional dimension of animal welfare is not known. The web-based suite of programs named Paraboss [14], which was developed as an aid in the management of the major parasite risks to sheep in Australia, provides another example of health and welfare related tools addressing the needs of farmers.

### 2.2. Benchmarking as an On-Farm Tool for Welfare Assessment

Problems of communication and technology transfer are at the heart of the deficiencies in welfare assessment procedures undertaken for compliance and assurance purposes in addressing the needs of farmers [15]. In the 1970s, agricultural extension officers began exploring alternatives to top-down communication to farmers as a means of gaining better engagement. In particular, methods then under development for improving business management through benchmarking were trialed [16]. These methods involve learning from the practical experience of businesses by comparing performance between businesses to identify best practices. More recently, versions of benchmarking as an approach to helping farmers manage welfare of their animals have been explored through health and welfare planning programs on organic farms in Europe [15], dairy cow comfort in North America [17], pig tail health in Sweden [18], and road transport practices in North America [19]. In 2013, a program of benchmarking grazing best management practice was introduced for beef cattle producers in the Fitzroy river basin of central Queensland [20]. The voluntary program includes an animal health and welfare module and uses self-assessment of management practices against industry standards. In its simplest form, benchmarking compares performance between industries to describe in statistical terms the range and average of outcomes achieved. In more detailed studies, it also identifies business practices associated with superior performance as exemplars that lower performing business might adopt. Benchmarking programs in a range of cereal grains and horticulture industries have proven highly successful for identifying best practices used by leading producers that enable fellow producers to improve performance [16,21]. These successes in cropping industries illustrate the potential for benchmarking to deliver benefits to livestock welfare management.

### 2.3. A Role for Risk Assessment in Welfare Management

Another approach to the development of tools for on farm management of animal welfare has been the use of risk assessments of the hazards that pose potential threats to welfare [22,23]. This strategy adopts the standard methodology of risk assessment that starts with identification of hazards then involves quantification of their likelihoods and potential impacts in terms of intensity, duration, and prevalence, in order to establish the need for monitoring and managing the risks. The European Food Safety Authority has developed risk assessment systems for a number of farm animal species and production systems for the dairy, chicken, meat, pig, beef cattle, and fish farming industries. A recent trend in agricultural benchmarking programs has been the combination of standard benchmarking procedures with risk assessment and risk management practices to provide both an aid to farm managers for the improvement of on-farm practices and a system for market assurance of farm practices and product quality [21]. Documentation of risk assessments, farm practices and monitoring procedures are important components of benchmarking and assurance schemes and provides a basis for internal and external auditing of the schemes. Cereal and horticultural industries have preceded livestock industries in adoption of this approach [24,25]; although use of risk assessment in livestock management is expanding. For instance, assessment of heat stress risk is a requirement for accreditation of feedlots in Australia [26].

## 3. A Unified Field Index for Managing Animal Welfare Performance On-Farm

To address the apparent shortcomings of current animal welfare assessment processes to assist farmers to manage welfare, we propose an approach for on-farm welfare management built around risk assessment, across enterprise benchmarking of welfare performance outcomes and external auditing. What follows is a provisional framework designed with a broad scope that might be applicable to all livestock sectors. The framework consists of assessment on a suite of domains that together with a process constitutes a new tool for livestock managers to monitor and improve the welfare of animals in their care. In addition, the tool generates records for internal and external audits that could provide assurance of welfare performance on individual farms and across a whole livestock sector. Audits help ensure integrity of the assessment process. We describe the framework as a Unified Field Index (UFI) for assessing welfare performance in the field (*i.e.*, on farm). We envisage its implementation through three levels:

- Level 1—occurs within the enterprise and is conducted by the livestock manager. It entails risk assessment, identification of the need for corrective actions, and key variables that require monitoring and recording. Results undergo periodic (e.g., yearly) internal review and self-audit to establish the need for modification and refinement. This level of a benchmarking program typically requires training and workshops to provide participants with the necessary skills [16].
- Level 2—is an external process that undertakes across-enterprise data analysis. The data analysis provides the confidential feedback to participants in the scheme that lets them benchmark their welfare performance against that of other producers. The analysis also identifies the practices of leading producers that contribute to their superior performance. It is the heart of the benchmarking process whereby new information and knowledge of superior practices is generated from on-farm experiences. Important outcomes of this analysis are the generation of industry-wide welfare statistics, as well as the identification of welfare performance goals and strategies that lower performing producers can adopt.
- Level 3—market assurance of practices and welfare outcomes can be provided through external audit of procedures undertaken in Levels 1 and 2.

**Figure 1:**
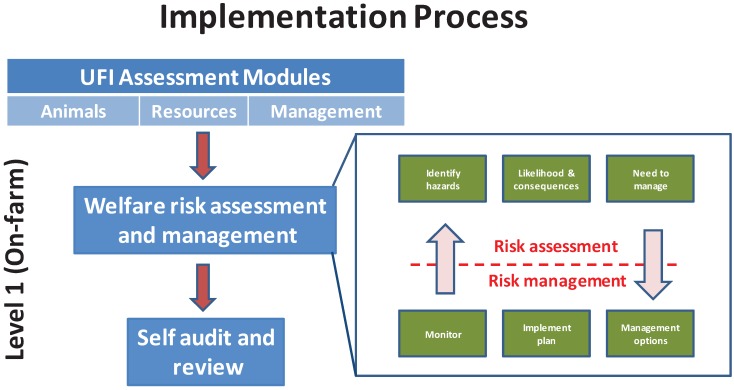
Level 1 (on farm) implementation of the UFI.

**Figure 2:**
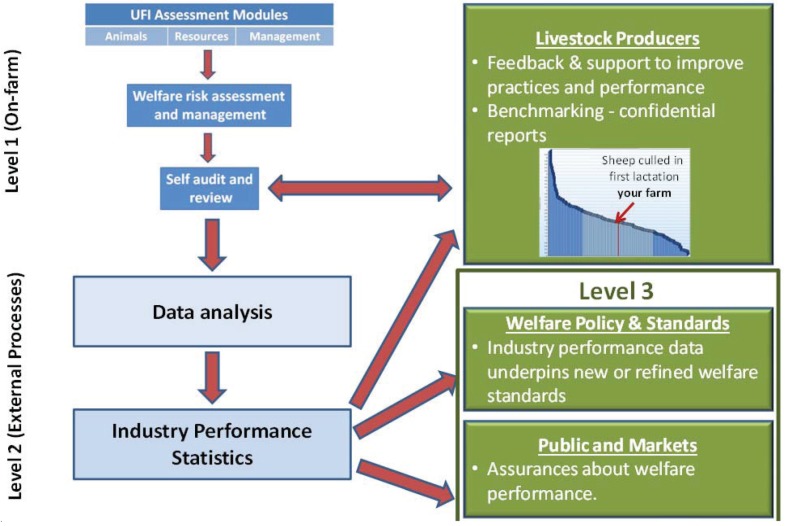
Schematic representation of implementation of the UFI.

We propose three assessment modules for the UFI that address animal, resource, and management based factors that are key input contributors to, or provide outcome indicators of welfare performance. Within each module are a number of domains as listed in Table 1. The domains provide the entry point for the risk assessment and for the development of monitoring and management strategies at Level 1 of the implementation process. Implementation of the UFI is presented schematically in Figure 1 and Figure 2.

**Table 1:** Assessment modules and domains of the Unified Field Index (UFI) for management of on-farm welfare performance with examples of classes and sub-classes within each domain. Classes and sub-classes need to be tailored to each particular species and production system.

Module	Domain	Class	Sub-class or measures
Animal	Behaviors	Abnormal	
		Social	Agonistic, Affiliative
		Self care	
	Health	Mortality	Number/rate, Causes, Number found dead, Number euthanized
		Morbidity	Numbers/rates, Causes
		Current status	Skeletal, Soft tissue, Coat/pelage, Demeanor
	Affect	Demeanor	Valence, arousal
		Avoidance behaviors	
	Production	Targets	Means, Variances
	Reproductive performance		
	Holistic attributes		
Resources	Feed	Food on offer	
		Quality	
	Water	Quality	
		Quantity	
	Climate	Risk of extremes	
	Social	Density	
		Group structure	
	Comfort	Bedding	
		Shelter	
	Hygiene	Housing	
		Floor	
		Animal body cleanliness	
		Health status of companions	
Management	Skills	Training/ experience	
		Attitudes	
	Husbandry practices	Methods	
		Analgesia	
		Euthanasia methods	
	Genetics management	Suitability of genetics for environment	
		Breeding objectives for welfare traits, disease resistance, temperament, conformation	Based on estimated breeding values
			Non-quantitative selection criteria
		Culling criteria	
	Records	Training	
		Husbandry	
		Vaccinations	
		Medications	
		Farm chemicals	
		Production	
		Health	
	Review and action protocols		

It is anticipated that co-ordination of a program for implementing the three levels of the UFI would require oversight by an industry body rather than by a regulatory authority. Indeed industry and farmer ownership of the tool and the outputs it generates is a key factor that differentiates the approach we propose here from conventional animal welfare assessments imposed on farmers though an authoritarian regulatory framework. Options for funding and management of such a program are not explored further in this paper.

## 4. Assessment Domains Included in the UFI

Our suggestion is that the assessment domains be largely consistent across species and production systems, although some minor modifications of the domains between species may be needed. Fine-tuning of the UFI to the nature of individual livestock species and their production systems would largely occur at the level of the class and sub-class of measures within each assessment domain. This tailoring to species and production system is analogous to the hierarchical design of the EU Welfare Quality program in which the four welfare principles (good feeding, good housing, good health, and appropriate behavior) are manifested through 12 welfare criteria which in turn are assessed through a suite of measures that are designed to be appropriate for each livestock species [27]. While the assessment domains in this general form of the UFI are broad in scope and number, it is anticipated that through the process of refinement at the industry level and implementation through the risk assessment procedure at the farm level, the number of domains to be monitored would be fine-tuned and reduced to the needs of each livestock industry, thus providing greater parsimony than is evident in the general form of the UFI. A limited number of measures would be required for each species or production system to ensure sufficiency of the process, especially to provide welfare performance statistics as described below. The use of the UFI as an assurance tool may involve meeting minimum standards within each domain. However, the integration of measures into one final score is not intended.

The domains are chosen to address the aspirational goals of the Five Freedoms [28] and the welfare principles and criteria of the EU Welfare Quality program. In addition, a number of management skills, management practices, and production criteria are included. These are described in a little more detail below. Electronic identification of individual livestock and the use of computerized records for individual animals are becoming widespread in livestock farming and create the opportunity for data collection and analysis to generate statistics of on-farm welfare performance in ways that have not been previously readily available to livestock managers. Many production variables are sensitive to stress and health status, and analysis of these variables across the whole flock or herd with particular emphasis on outliers and deviations from performance targets can provide information on welfare performance. Animal performance measures are therefore also included within the assessment domains of the UFI. The rapidly emerging electronic technologies for monitoring animals have the potential to assist farmers in the animal monitoring phase of Level 1.

### 4.1. Animal Module

#### 4.1.1. Behavior Domain

*Abnormal Behaviors*: Animals display a range of abnormal behaviors when stressed or when exposed to adverse environmental conditions such as heat. Stereotypic (repetitive, rhythmic) behaviors are common in intensively-housed animals with impoverished environmental conditions. Fearfulness and other indicators of adverse reactivity of animals to the presence of humans that can be indicative of poor animal handling practices sit in this class.

*Social behaviors*: Social behaviors like mutual grooming and play are important indicators of the social health of social species like cattle, sheep, pigs, and chickens.

*Self-care*: Self-care is reduced in animals stressed by poor nutrition, ill health, and social bullying.

#### 4.1.2. Health Domain

*Mortality*: Raw data on mortality can be criticized as being a worst case outcome of welfare management. Nonetheless, deaths do occur in well-managed livestock enterprises and the information that death rates provide about welfare management should not be ignored by livestock managers. Data on the numbers of animals found dead versus the number of animals euthanized is a valuable indicator of the level of monitoring and intervention to prevent suffering in moribund animals.

*Morbidity*: Disease compromises welfare, thus data on disease prevalence is central to effective disease control and good welfare management.

*Current status*: This domain provides the basis for visual assessment of the general health of the individual and the group. These measures are examples of ones that would be used in external audit for a snapshot of the flock or herd as well as during ongoing monitoring by the livestock manager.

#### 4.1.3. Affect Domain

Qualitative behavioral assessment has been found to be a useful method for assessing affective states of livestock on farm [29,30,31]; however, the methodology is not currently suitable for application by individual observers such as livestock managers. Nonetheless, the assessment of demeanor or behavioral expression as used in clinical assessment of animals by veterinarians and by skilled livestock handlers such as pen riders in feedlots are two examples of visual assessments of behavior that most likely provide information on the emotional status of animals associated with ill health. Standardization and refinement of these measures and the development of new field-based measures of affect are needed.

#### 4.1.4. Production Domain

Analysis of performance against production targets, especially the identification of the number of outlier animals, provides information on the level of management of the enterprise. Increased risk of disease and death in animals below the group mean for growth, body weight, and body condition is recognized in a range of species including lambs [32], dairy cows [33], pigs [34], and rainbow trout [35]. While performance against targets is not always informative of welfare, in combination with other measures, it can help provide a picture of welfare management of the enterprise. The value of these data is greatly enhanced through benchmarking. The domain does not equate good production with good welfare but recognizes that variation in production performance can be an indicator of potential welfare problems.

#### 4.1.5. Reproductive Performance Domain

Reproductive performance is highly sensitive to stressors and diseases that affect welfare, as well as being fundamental to the profitability of the breeding enterprise, and is strongly influenced by the quality of management that animals receive and the suitability of the genotype for its environment. This class could be included with production, but is separated to emphasize the very high importance of good management of reproduction for achieving good welfare outcomes [36].

#### 4.1.6. Holistic Domain

This assessment domain is a measure of naturalness and acknowledges philosophies which value “whole-of-animal” and “whole-of-production-system” attributes of welfare. Examples of holistic measures might include the ability of turkeys to reproduce by natural mating, and the opportunity for animals to express highly motivated behaviors such as nesting, rooting, and wallowing in pigs, dust bathing and scratching in poultry, and grazing in ruminants. It might be argued, however, that these examples are component measures that would be better captured in the behavioral domain than true holistic measures.

### 4.2. Resource Module

#### 4.2.1. Feed Domain

Feed quality, quantity, ease of access, and suitability for the metabolic needs of the animals are important resources the animal needs.

#### 4.2.2. Water domain

Includes quality, quantity, and ease of access.

#### 4.2.3. Climate Domain

Includes the range of climatic variables animals are exposed to with particular emphasis on extremes.

#### 4.2.4. Social Resources Domain

Social behaviors are listed within the animal module. This domain addresses social conditions imposed on the animal such as stocking density, group structure and access to companion animals.

#### 4.2.5. Comfort Domain

This domain addresses indoor and outdoor infrastructure including bedding, availability of shelter, and protection from climatic extremes that affect comfort of animals.

#### 4.2.6. Hygiene Domain

Hygiene conditions influence exposure to disease pathogens and environmental organisms that influence health and welfare. Management of diseased animals such as use of hospital pens in order to reduce exposure of an individual to infectious conspecifics is considered in this domain.

### 4.3. Management Module

While access for animals to the environmental resources listed above is controlled by management practices, the Management Module addresses non-resource aspects of animal management as well as business practices that impinge on animal welfare. It also addresses some of the process elements that are necessary for the program to be effective.

#### 4.3.1. Skills Domain

Addresses whether stockpersons have appropriate skills training (or experience) and appropriate attitudes for working with animals.

#### 4.3.2. Husbandry Practices Domain

This class addresses the methods used for husbandry practices, ages when the practices are performed, and whether analgesia is used for painful procedures. Methods used for euthanasia are documented also.

#### 4.3.3. Genetics Management Domain

The impact of genetic practices on welfare of livestock has not been addressed in previous welfare assessment schemes, yet many livestock welfare problems are closely linked to the genetics of the animals [37]. So not only do genetic practices contribute to many welfare problems, they can also provide a partial solution to some of these problems. This domain addresses issues such as: (1) suitability of the genotype for the production environment; (2) ways animals are selected for breeding purposes including use of estimated breeding values (EBVs) and genetic tools such as the polled gene marker test in cattle [38]; (3) the weight given to welfare traits within quantitative multi-trait breeding objectives; and (4) non-quantitative criteria (e.g., visual classing criteria) used for selecting breeders. Culling criteria, culling numbers, and culling age can provide important information on welfare performance of the enterprise, as defects and poor performance of individuals that result in them being culled are often caused by or related to poor welfare.

#### 4.3.4. Records Domain

Records are essential for self-audit and internal review, as well as for providing data for benchmarking and external audit, and are also required in many jurisdictions to substantiate compliance with regulations around use of agricultural and veterinary chemicals, product declarations and occupational health and safety.

#### 4.3.5. Review and Action Protocols Domain

Success of the program depends on periodic internal review of data and of management processes. Review process, outcomes, and actions taken need to be documented.

## 5. What Is New with the Unified Field Index?

Current welfare assessment procedures undertaken for compliance and assurance purposes are largely based on the concept of standards that have been derived from research or consensus of expert opinion. Standards are accompanied by the risk that they are interpreted as all or nothing thresholds that only need to be exceeded but not continually improved upon [7]. In contrast, the UFI introduces the concept of welfare performance as a continuous trait (or suite of traits) or attributes of an enterprise that is measured through benchmarking of performance across enterprises within an industry. While yet to be substantiated by evidence, it is anticipated that feedback from benchmarking will create an incentive for continuous improvement in welfare performance and furthermore, that livestock farming industries will feel greater ownership of the methodology for monitoring welfare performance than of welfare standards imposed on them through top-down, authoritarian, external assessment schemes.

The UFI also utilizes data that are available through individual electronic identification of animals and computer based animal records to generate information on welfare performance from conventional livestock performance measures like growth rates, reproduction rates, culling statistics, and disease prevalence. Ideally, only relatively minor modifications would be needed to software currently used for managing livestock performance data in order to record additional welfare related variables. The UFI proposes that standardized industry-wide statistics of welfare performance be generated from this on-farm data for feedback through benchmarking processes to individual producers (Figure 2) and for public reporting of industry performance. While the suite of welfare performance statistics is likely to differ between industries, data on mortality and morbidity have been largely overlooked as measures of welfare by point-in-time welfare assessment programs such as Welfare Quality. Although death is recognized as a blunt measure of animal welfare, it nonetheless is a foundational measure of welfare performance of an enterprise. The science of medical statistics was born when John Graunt began analyzing the London Bills of Mortality during the Great Plague of London in 1665–1666. These types of statistics including the causes and numbers of deaths, illness, therapeutic treatments, and culling provide a starting point for the description of welfare performance of a livestock enterprise. Many of these statistics are currently estimated by livestock managers because they are important drivers of profitability. What is new in the UFI is the use of these statistics to measure welfare performance. Better defined statistics of welfare performance also have the potential to provide some new traits for genetic evaluation programs, although some welfare traits such as resistance to internal parasites based on worm egg counts are already well recognized and used in breeding programs [39]. It is anticipated that across-enterprise analysis of welfare performance will provide valuable knowledge to aid the development of regulatory standards for farming practices and animal welfare.

The UFI places greater emphasis on management practices than previous assessment schemes. Large bodies of scientific research have identified the impact of training, attitudes, husbandry practices, use of analgesics, euthanasia methods, and genetic practices on the welfare of animals; however on-farm assessment of these practices has previously focused on compliance with laws and regulations rather than recognizing the scope for farmers to adopt improved practices. Genetic practices are a particular case in point, where there is considerable scope for farmers to implement breeding programs such as selection for resistance to blow fly strike in sheep that improve welfare outcomes [40].

Through the use of self-assessment of welfare risks and self-monitoring of welfare performance, the UFI is largely non-prescriptive of the methods and practices farmers should use to achieve favorable welfare performance. Rather, through appropriate measurement and monitoring, it proposes farmers can generate their own understanding of good practices on their farm and learn from the achievements of similar enterprises. Indeed, benchmarking schemes and risk assessment protocols have the intent of being educational and informative for the user and at the same time empowering the user to be a better manager [16].

The UFI incorporates elements of each of the three perspectives for assessing animal welfare as noted in the introduction: biological functioning, emotional states, and naturalness. The first two share with the UFI a strong dependence on outcome measures based on animals, while the third has reliance both on outcome measures and input measures, especially environmental conditions that are predictors of adverse welfare outcomes. What the UFI may bring to the practice of welfare assessment is a stronger acknowledgment of the role of management practices as predictors of welfare outcomes and recognition of the role of dynamic management procedures in the continual improvement of welfare performance.

Thus, the UFI represents a change from the authoritarian imposition of animal welfare standards on farmers for compliance and some market assurance purposes on a number of counts. It is designed to provide a mechanism for farmers to improve the welfare of their livestock. It is non-prescriptive in the description of good welfare. Rather it sees knowledge and understanding of the levels of welfare achieved by industry as emerging from the data acquired from farming enterprises. It acknowledges the experience of farmers as a source of new knowledge on management practices that lead to superior welfare performance. It increases ownership and responsibility for welfare performance by farmers. In these respects, it follows the model for benchmarking of production performance used in many agricultural industries. Its primary purpose is to assist farmers to improve animal welfare outcomes in their businesses and the tool may, when further developed, be able to underpin some market assurance schemes. A potential outcome of this approach is the development of a stronger animal welfare culture within farm animal industries and an ability for the industries to demonstrate that they foster this culture. The UFI is not a competitor to welfare assessment procedures undertaken for compliance purposes, but a complementary approach to the more general goal of improving farm animal welfare.

## 6. Limitations of the UFI

As a prototype, the UFI is yet to be tested as a method of empowering livestock managers to achieve better welfare performance on their enterprises. The elements of self-assessment and self-monitoring may fail to satisfy consumers of the integrity of measures of welfare performance despite the proposed inclusion of external auditing processes. While the UFI may fail to fulfill market assurance purposes, the benchmarking and welfare management goals of Levels I and II on their own should be valuable. By introducing the concept of welfare performance, the UFI might face resistance from advocacy groups and consumers by being seen as an attempt to reframe the concept of welfare rather than as an attempt to introduce a new approach for improving the management of welfare. Some livestock industries might consider welfare performance statistics to be too sensitive or too readily subject to misinterpretation for public disclosure. On the other hand, a focus on statistics as a summary of welfare performance might overshadow for some farmers the importance of identifying and managing individual animals in a poor welfare state. In its present form, the UFI makes no attempt to combine or integrate measures across domains in order to generate an overall score or assessment of welfare performance and does not consider any trade-offs that might occur between measures in the assessment process. And finally, like other welfare assessment procedures, the UFI will remain encumbered by limitations in our knowledge of and ability to assess affective states of farm animals in the field.
